# Affect-Laden Imagery and Risk Taking: The Mediating Role of Stress and Risk Perception

**DOI:** 10.1371/journal.pone.0122226

**Published:** 2015-03-27

**Authors:** Jakub Traczyk, Agata Sobkow, Tomasz Zaleskiewicz

**Affiliations:** Faculty in Wroclaw, University of Social Sciences and Humanities, Wroclaw, Poland; University of Chicago, UNITED STATES

## Abstract

This paper investigates how affect-laden imagery that evokes emotional stress influences risk perception and risk taking in real-life scenarios. In a series of three studies, we instructed participants to imagine the consequences of risky scenarios and then rate the intensity of the experienced stress, perceived risk and their willingness to engage in risky behavior. Study 1 showed that people spontaneously imagine negative rather than positive risk consequences, which are directly related to their lower willingness to take risk. Moreover, this relationship was mediated by feelings of stress and risk perception. Study 2 replicated and extended these findings by showing that imagining negative risk consequences evokes psychophysiological stress responses observed in elevated blood pressure. Finally, in Study 3, we once again demonstrated that a higher intensity of mental images of negative risk consequences, as measured by enhanced brain activity in the parieto-occipital lobes, leads to a lower propensity to take risk. Furthermore, individual differences in creating vivid and intense negative images of risk consequences moderated the strength of the relationship between risk perception and risk taking. Participants who created more vivid and intense images of negative risk consequences paid less attention to the assessments of riskiness in rating their likelihood to take risk. To summarize, we showed that feelings of emotional stress and perceived riskiness mediate the relationship between mental imagery and risk taking, whereas individual differences in abilities to create vivid mental images may influence the degree to which more cognitive risk assessments are used in the risk-taking process.

## Introduction

Normative decision theory assumes that people estimate risk and make choices under uncertainty in a rational way, i.e., they weigh outcomes by probabilities in order to calculate the expected utility associated with all the alternatives [1]. However, numerous studies conducted in the field of behavioral decision research have demonstrated that people violate assumptions of the expected utility model and make their choices relying on their gut feelings instead of calculating the quantitative parameters [2,3].

In real-life, or non-monetary risky decision dilemmas, information about probability is rarely available. Therefore, when considering whether to have sex with an unknown partner or to try drugs which pose a threat to our health, we often spontaneously imagine the consequences of our choices and make decisions on the basis of experienced emotions driven by the risky situation instead of thinking in terms of probability [4,5]. What is more, even if the objective value of probability is known, experiencing strong anxiety might lead a decision maker to ignore or dismiss this relevant information [6]. The main thesis of the present paper holds, with the above line of argument, that emotional reactions (e.g., stress) driven by vivid images of consequences resulting from dangerous behavior, influence subsequent risk perception and risk taking.

### The role of emotions in risk perception and risk taking

**Fig 1:**
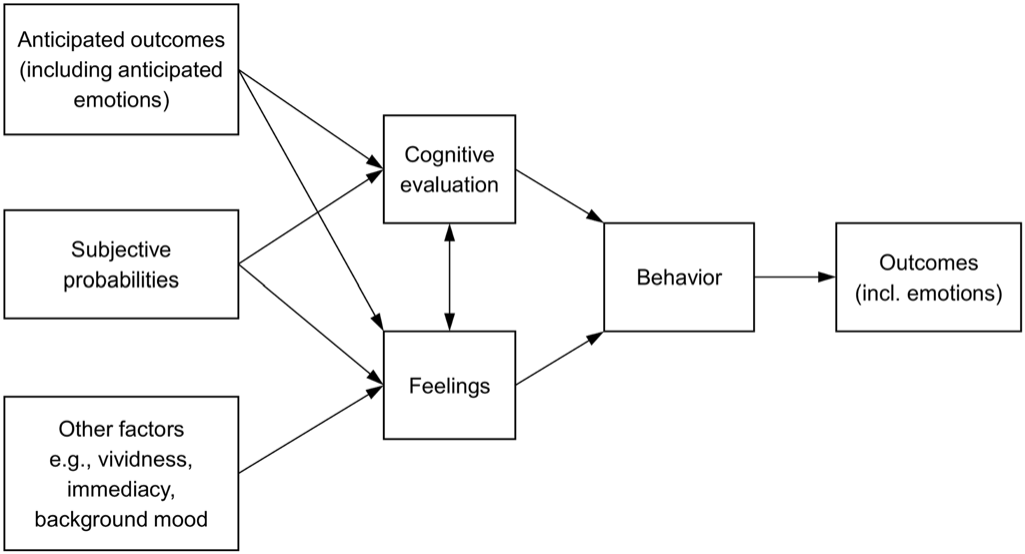
The risk-as-feelings model adapted from Loewenstein et al. [4].

The notion that emotional reactions play a crucial role in decision processes was highlighted in such models as the affect heuristic [2] and the risk-as-feelings hypothesis [4]. The authors of the latter theoretical approach postulate that “responses to risky situations (including decision making) result in part from direct (i.e., not cortically mediated) emotional influences, including feelings such as worry, fear, dread, or anxiety” ([4] p. 270). The model, as shown in Fig. 1, assumes an interplay between affect and cognition in producing behavior under risk. Moreover, it posits that some emotional factors indirectly influence risky choices with only little or even without cognitive control as in panic reactions to threatening stimuli. Similarly, we suggest that emotional reactions to risk might evoke from vivid and intense mental images.

The risk-as-feelings hypothesis proposes that even subtle affective reactions associated with a risky situation influence our subsequent behavior in the context of risk and uncertainty. Importantly, in contrast to other theoretical propositions, it posits that emotions cannot be reduced only to states that are experienced when the outcomes of a decision are already known. In other words, people’s behavior in risky situations is strongly determined by anticipatory emotions (e.g., fear or dread) that are driven by both the characteristics of the risk and different individual dispositions (e.g., trait-anxiety).

Emotions, as discussed above, not only impact and direct risky behavior but also interact with the cognitive analysis of risk. For example, processing of the numerical characteristics of a decision problem (its outcomes and probabilities) might be distorted by intense feelings associated with emotional stress produced by vivid mental representations of a risky situation. Hsee and Rottenstreich [6,7] demonstrated that affect-rich options in comparison to affect-poor options led to lower sensitivity to variations in quantitative aspects of a stimulus (magnitude, scope) and probabilities. On the other hand, research in neurological patients who are not able to process affective information normally showed that they preferred disadvantageous and risky (high reward/high loss) alternatives [8,9]. One possible interpretation of this effect might be that these types of patients are very prone to accept risk because their mental images of risk are not coupled with emotional states. In the same vein, we argue that intensive negative emotions that influence and direct risk perception and risk taking might stem from the mental imagery of the negative consequences associated with dangerous behavior. Earlier research has preliminarily demonstrated an association between affect and risk perception for both environmental and financial risks [10,11]. In our research, we examine, in a more detailed way, the relationship between affect-laden mental imagery, risk perception and risk taking.

### Affect-laden imagery in risk perception and risk taking

Stephen Kosslyn [12] suggested in his seminal book “Image and Brain” that anticipatory emotions are strongly related to mental imagery. This has been empirically confirmed by Holmes and Mathews [13], who documented that people who imagine unpleasant events reported more anxiety than those who were thinking about the verbal meaning of the descriptions of these events. Moreover, Öhman and Mineka [14] argued that feelings are more sensitive to imagery than to narration because language evolved later than such basic emotions as fear. This latter argument may be of special importance in the context of studying risky behaviors because, as we argued earlier in this paper, both risk judgments and risk preferences are strongly associated with anxiety. In this sense, emotional reactions evoked by mental imagery could mediate the perception of risk.

Earlier studies on risk perception have demonstrated that the way people perceive the risk associated with a nuclear waste repository was strongly related to their verbal imagery of this risk [15]. It has been shown that almost all participants who rated their images as negative said that they would vote against a repository at Yucca Mountain, revealing a strong bias toward risk-averse action. Peters and Slovic [16] found a significant and positive correlation between affect associated with mental images related to nuclear power and the nuclear support index. However, our approach differs from the studies cited above in several ways. First, our participants were asked to visualize their images of risk consequences and not to list verbal associations. Second, we used not only declarative but also psychophysiological measurements of the intensity of people’s affective reactions to their risk images. Next, we explored the influence of anticipatory (evoked by creating mental images of risk) emotions on both risk perception and risk taking. Finally, the aim of our research project was not only to analyze the general process of risk perception and risk taking but also to examine the role of individual differences in creating vivid and intense mental images of risk (as proposed in the risk-as-feelings hypothesis [4]). We tested how differences in the intensity of imagining the consequences of risk (understood as individual tendencies to create vivid visual mental images) moderate the association between risk perception and risky behavior.

### The overview of the studies and research hypotheses

To summarize, on the basis of the risk-as-feelings model, three studies were carried out to test the following hypotheses. In Study 1, we hypothesized that risk-related thoughts (i.e., anticipated outcomes of risk; see Fig. 1) would be related to both stress (i.e., negative feelings) and risk perception (i.e., cognitive evaluation) leading to a lower willingness to take risk (i.e., risk-taking behavior). Participants were asked to report the first three thoughts related to a stated risk and then to indicate their feelings of stress, risk perception and willingness to take risk (a similar procedure for studying the role of affect in risk perception and risk taking was successfully used before, see [17]). The aim of Study 2 was to replicate the findings of Study 1 with the use of a physiological measure of the stress level (blood pressure). We hypothesized that subjective stress levels and risk perception would serially mediate the relationship between the vivid and intense images of negative risk consequences and risk taking. Finally, in Study 3 we predicted that the objective intensity of mental imagery measured by brain activity (EEG) would be related to risk-taking behavior. Furthermore, our model assumed that individual differences in the intensity of vivid negative images of risk would moderate the relationship between risk perception and risk taking.

### Ethics statement

Experiments conducted in the present research were approved by the Ethics Committee at the University of Social Sciences and Humanities in Wroclaw. Subjects signed written informed consent prior to participation (Study 2 and 3) or the consent was given by clicking a designated button starting a computerized procedure (Study 1).

## Study 1

### Method

#### Participants

Sixty undergraduate students (30 females) aged 24.7 on average (*SD* = 5.9) participated in this study for credit points. Their participation was voluntary, anonymous and in agreement with the guidelines of the Ethical Committee.

#### Materials and procedure

Participants were instructed to read ten risky scenarios containing short descriptions of risky situations that covered five risk domains (financial, health/safety, recreational, ethical and social) identified earlier by Weber and colleagues in their research [18]. Two independent scenarios were prepared for each risk domain. Below, one example of a health risk scenario is presented:

“For some time, you’ve been suffering from some health problems. You should have a medical examination, but it is very unpleasant and requires staying in the hospital for several days. Unfortunately, you are preparing a very important project at work now, and you decide to reschedule the examination. Your physician warns you against postponing this examination because, if you really are ill, this disease can develop very quickly and in a few weeks it will be too late for treatment.”

Then, participants were asked to imagine that they were involved in these scenarios and to write down the first three thoughts (either positive or negative) associated with the situations presented to them (only data from participants who produced three risk-related thoughts were used in further analysis). Afterwards, they assessed: (1) the valence of their feelings (emotions) evoked by each association using a one-dimensional 5-point scale (i.e., risk-related thoughts, from -2 –“negative” to 2 –“positive”), (2) the riskiness of the situation (i.e., risk perception, 10-point scale from 1 –“not risky at all” to 10 –“extremely risky”), (3) their willingness to behave in the same way as the person in the scenario (i.e., risk taking, 5-point scale from 1 –“definitely not” to 5 –“definitely yes”) and (4) the stressfulness of each situation (i.e., stress, 10-point scale from 1 –“not stressful at all” to 10 –“extremely stressful”). The reliability of measures used in this study was assessed by intraclass correlation coefficients (ICC). Satisfactory reliability was found for risk-related thoughts (ICC = .80), risk perception (ICC = .68), risk taking (ICC = .70) and stress (ICC = .81). Additionally, two self-report measures were used to diagnose individual differences in trait anxiety level (the STAI; [19]) and risk propensity (the SIRI scale; [20]).

### Results

#### Analytic strategy and rationale

**Fig 2:**
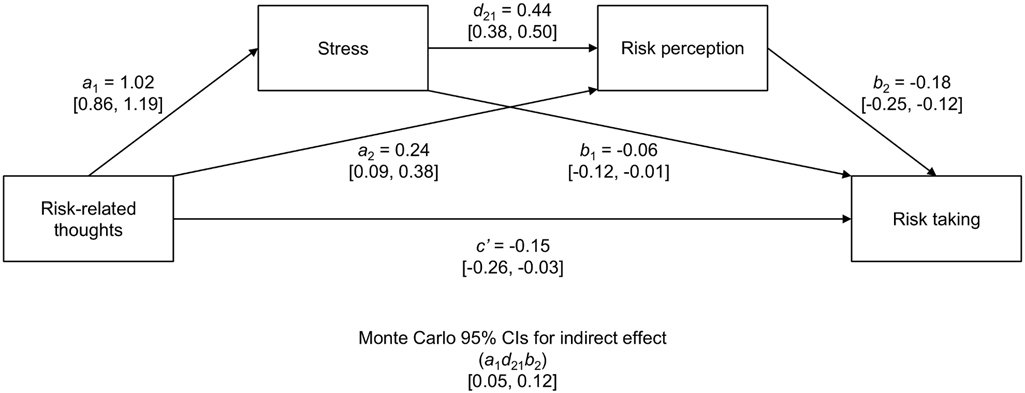
Multiple mediator model in Study 1. Unstandardized regression coefficients, corresponding to the serial multiple mediator model linking risk-related thoughts (X) and risk taking (Y) via stress level (M_1_) and risk perception (M_2_). The model is adjusted for individual differences in stimulating-instrumental risk taking (SIRI) and trait-anxiety (STAI). STAI was related to Risk-related thoughts, *r* = .49, *p* <. 001; Stress, *r* = .44, *p* <. 001; Risk perception, *r* = .34, *p* = .007. SIRI was related only to Risk taking, *r* = .36, *p* = .004 (two-tailed). Lower and upper limit of 95% Monte Carlo confidence intervals are given in square brackets.

We applied a serial multiple mediator model to determine whether the emotional valence of risk-related thoughts (predictor variable, X) exerts an effect on risk taking (outcome variable, Y) through changes in other variables such as stress and risk perception (mediator variables, M_1_ and M_2_) situated in a causal path between X and Y [21]. In this method, a total effect of X on Y is partitioned into two components (see Fig. 2 for details): direct (X regressed on Y) and indirect (paths: *a*_1_ *b*_1_ + *a*_2_ *b*_2_ + *a*_1_ *d*_21_ *b*_2_). We were especially interested in estimating a two-mediator indirect path model (*a*_1_ *d*_21_ *b*_2_), which assumes that mediator variables are related, even when controlling for the influence of a predictor variable on these mediators. In other words, the direct and indirect effects of X on Y are investigated by modeling a process in which X influences M_1_, which in turn influences M_2_, which finally causes Y. In comparison to other multiple mediator models, the serial multiple mediator model is supposed to be applied to causally related data. Therefore, the assumption of no correlation or causal relationship between mediators is rejected a priori [21] (i.e., variables can be correlated within-subjects). To construct the lower and upper limits of the 95% confidence interval for the indirect path (*a*_1_ *d*_21_ *b*_2_), we adapted the Monte Carlo simulation method [22]. In this method, an empirical approximation of an *a*_1_ *d*_21_ *b*_2_ sampling distribution is generated by simulating *k* random draws from the three normal distributions for each of the indirect effect regression estimates (i.e., *a*_1_, *d*_21_ and *b*_2_) and associated standard errors. When the estimated confidence interval does not encompass a value of zero, we can conclude that the indirect effect is significant. This means that a two-mediator path significantly mediates the link between X and Y.

In each study reported in the present project, analyses were performed on non-aggregated data from all the participants and risky scenarios/risk domains. Such a data structure may lead to violation of the non-independence assumption—risk ratings could be correlated within subjects and items (risky scenarios/domains), what inflates the type I error as a consequence. To resolve the non-independence problem that stems from having multiple responses by the same subject or for a risk domain, regression coefficients were estimated by applying a mixed-effect model [23]. In this approach, we accounted for by-subject and by-item variation in overall risk taking propensity by including subjects and items as random-intercept effects. In other words, we assumed that an intercept in fitted models can vary across different participants and risk domains.

Finally, we used a likelihood ratio test to compare the serially mediated model fit (including two mediators: M_1_ and M_2_) with the baseline model fit (including only X). We assumed that introducing mediator variables would improve overall model fit, evidencing the importance of both emotional and cognitive factors in predicting risk-taking behavior.

#### Multiple-step multiple mediator model of risk-related thoughts, stress, risk perception and risk taking

We used the lme4 software package [24] supplied in the R statistical environment [25] to estimate fixed regression coefficients for the serial multiple mediator analysis. In each model, subjects and risky situations were included as crossed random-intercept effects. Additionally, models with risk taking ratings as a dependent variable were adjusted for covariate variables (SIRI and STAI—correlations between these covariate variables and other measures are presented in Fig. 2 caption). The significance of the indirect *a*_1_ *d*_21_ *b*_2_ effect was tested by generating 95% Monte Carlo confidence intervals based on 100,000 simulated samples.

In order to simplify the interpretation of results, data concerning the emotional valence of risk-related thoughts were recoded so that higher values represented more negative associations (from -2 –“positive” to 2 –“negative”, *M* = 0.83, *SD* = 0.98). A repeated measures ANOVA with ten risky scenarios as a within-subjects factor was performed to compare the mean emotional valence of risk-related thoughts produced in these scenarios to neutral ratings (0). The analysis revealed a main effect of the within-subjects factor (i.e., risky scenarios), *F*(10, 590) = 22.324, *p* <. 001, η^2^ = .275. Further post-hoc analyses with Bonferroni-Holm correction for multiple comparisons showed that the mean valence of risk-related thoughts was negative and differed significantly from neutral ratings for every risky situation (two-tailed *p*-values adjusted for multiple comparison ranged from *p* <. 001 to *p* = .019 depending on tested scenario). As expected, we found that more negative associations of risk consequences led to higher stress (*a*_*1*_ = 1.02), which in turn influenced a higher assessment of risk (*d*_21_ = 0.44) and, as a consequence, resulted in a lower willingness to take risk (*b*_2_ = -0.18). Critically, the indirect effect via both mediators (*a*_1_ *d*_21_ *b*_2_) was significant, 95% CI [0.05, 0.12], suggesting that stress and risk perception mediated the relationship between mental images of risk and risk taking. Moreover, even when controlling for the indirect effect, more negative risk-related thoughts led to a lower willingness to engage in risky behavior (*c*’ = -0.15).

Finally, we compared the baseline model fit (including only risk-related thoughts as a predictor) to the mediating model fit (additionally incorporating mediator variables: stress and risk perception). The log-likelihood ratio test revealed better fit for the serially mediated model, χ^2^(2) = 65.072, *p* <. 001, indicating that inclusion of stress and risk perception improved the model predicting risk-taking behavior.

### Discussion

In accordance with the risk-as-feelings hypothesis [4], behavior in a risky situation might be interpreted as the result of interplay between different cognitive and emotional factors. Our Study 1 demonstrated that the emotional valence of risk-related thoughts induced by thinking about a risky situation is generally negative. Moreover, these negative emotional mental images induce the feeling of stress that, together with a subsequent assessment of riskiness, improves the fit of the model to the data and mediates the relationship between the mental representation of risk and risk taking. In this manner, our data support recent findings presented by van Gelder, de Vries and van der Pligt [17] who used structural equation modeling to demonstrate that negative affect significantly predicts risk perception and risky behavior. However, as opposed to the study of van Gelder et al. [17], our main question of interest was not only related to the associations between negative affect and risk perception per se, but also to the role of mental imagery which evokes affective and cognitive responses. This helps us to better understand the impact of affect on risky behavior. Therefore, our results seem to provide evidence for a substantial role of emotional processes in risky behavior and offer initial empirical support for the risk-as-feelings model.

However, the valence of emotional reactions evoked by associations with risk is not the only crucial factor for risk-taking behavior. The vividness of these associations may also play an important role in this process. In our next study we introduced a modification in our experimental design to examine whether the vividness and intensity of mental images of risk consequences would be related to an enhanced stress level, increased risk perception and a lower willingness to accept risk.

The additional aim of Study 2 was to test the hypothesis concerning the causal relationship between imagining risk consequences and stress. In other words, this study examined the assumption that thinking about risky situations evokes feelings of stress. However, it is also plausible that experienced stress reactions are not task specific but are rather produced by a laboratory setting. The context of the experiment leads to an elevated stress level independent of imagining risk consequences. We addressed this problem in Study 2. We hypothesized that imagining negative consequences of risk would causally lead to an increase in stress reactions as measured by both subjective affect assessment and changes in blood pressure. Moreover, objective psychophysiological changes in blood pressure should be task-specific, which means that they would be observed only when participants are instructed to imagine risk consequences and not be observed in baseline recordings.

## Study 2

### Method

#### Participants

To avoid a possible influence from gender differences in blood pressure level [26] only females were invited to take part in the study. Twenty-two female undergraduate students (average age: 25.3, *SD* = 7.2) without diagnosed arrhythmia or other cardiovascular diseases gave their informed consent to participate in the study in exchange for credit points. Participants were asked to restrain from physical activity, smoking, drinking coffee or energy drinks and eating large meals for two hours before the study. Participation in this study was voluntary, anonymous and in agreement with the guidelines of the Ethical Committee.

#### Materials and apparatus

Participants listened to 30-second risky scenarios taken from the set used in Study 1. Each scenario was recorded with text-to-speech software to reduce the emotional meaning conveyed by prosody of speech. Each participant listened to five randomly selected scenarios covering five risk domains (the same as in Study 1). One additional scenario was applied as training material to familiarize participants with the procedure.

Two measures of stress evoked by imagining negative risk consequences were used: (1) systolic and diastolic blood pressure registered with a digital blood pressure monitor TMA-880 (error +/- 3 mmHg) manufactured by TechMed (a similar apparatus and similar testing procedures were used in previous studies [27]) and (2) state of positive (PA) and negative affect (NA) measured with the Positive and Negative Affect Schedule (PANAS, [28]) that consists of 20 adjectives describing different feelings and emotions. The PANAS was widely used in previous studies on psychological stress to assess people’s affective reactions [29].

#### Procedure

Participants were seated individually in a dimly lit room and asked to relax for five to ten minutes with their eyes closed. Then, eight measurements of pre-test baseline blood pressure were performed in 30-second intervals. This was followed by administering the PANAS to measure a pre-test baseline affective state. Afterwards, participants listened to one training scenario and five test scenarios presented in a random order. For each scenario, participants were instructed to imagine, for the next 30 seconds, all the negative consequences of the described risky behavior. Meanwhile, one reading of blood pressure was taken. Having completed each of the visualization phases, participants evaluated: (1) the intensity and vividness of the mental images evoked by each risky scenario (from 1 –“not intense and not vivid at all” to 10 –“very intense and vivid”), (2) the stressfulness of each scenario (i.e., stress, from 1 –“not stressful at all” to 10 –“very stressful”), (3) the riskiness of each scenario (i.e., risk perception, from 1 –“not risky at all” to 10 –“extremely risky”) and (4) their willingness to take the same risk as the person shown in the scenario (i.e., risk taking, from 1 –“definitely not” to 10 –“definitely yes”). The next scenario started immediately after answering all the items listed above. Contrary to Study 1, participants did not verbally report their risk-related thoughts.

In the last stage of the study, participants completed the PANAS once again to measure the post-test change in their emotional state evoked by imagining the negative consequences of the risk. Then, to obtain post-test baseline blood pressure, six readings of blood pressure in 30-second intervals were taken while participants were relaxing with their eyes closed. Additionally, to control for individual differences in risk-taking propensity, participants completed the SIRI scale [20] in counterbalanced order. Half of them did so at the beginning and the other half at the end of the study.

### Results

#### Data reduction

Blood pressure measurements can be distorted by stress derived from a new laboratory setting (an unfamiliar laboratory equipped with psychophysiology apparatus might lead to an elevated baseline stress level) and individual post-stress recovery period (an elevated blood pressure level might remain even after the termination of the stressor). To control for these possible confounds, all steps in data reduction were performed according to the guidelines for analyzing cardiovascular data in stress research [30]: (1) twenty measurements of systolic (SY) and diastolic (DY) blood pressure for each participant were transformed into Mean Arterial Pressure (MAP = 2/3*DY + 1/3*SY); (2) five measurements, the first three from the first baseline (at the beginning of the study), one from the training scenario and the first from the second baseline (at the end of a study), were removed from further analysis; (3) the remaining 15 measurements were z-scored for each participant; (4) five measurements from the first and five from the second baseline were averaged separately and (5) five measurements from the visualization phase (five risky scenarios) served as psychophysiological indicators of stress evoked by a mental representation of risk consequences. The benefit of such data reduction approach is a better control for both (1) possible confounds related to laboratory stress induction and (2) intra-individual differences in a blood pressure response.

#### Changes in the experience of imagery-driven stress levels

**Fig 3:**
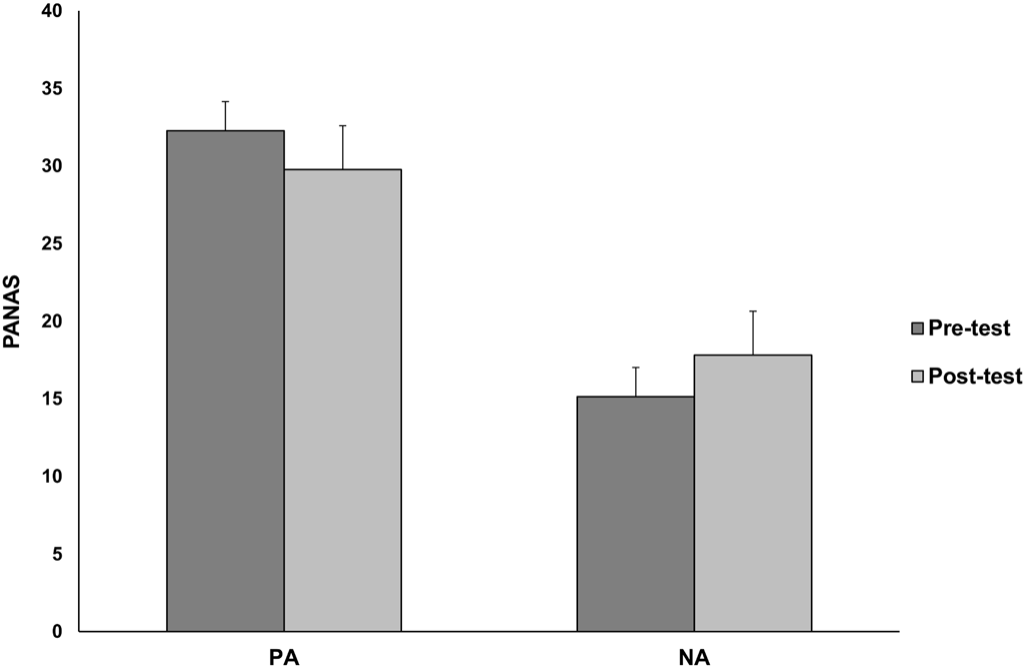
Mean PANAS scores before (pre-test) and after (post-test) imagining negative risk consequences in Study 2. There was a significant decrease in positive affect (PA; *p* = .018) and an increase of negative affect (NA; *p* = .032) led by imagining the negative consequences of risky situations. Error bars represent 95% confidence intervals.

**Fig 4:**
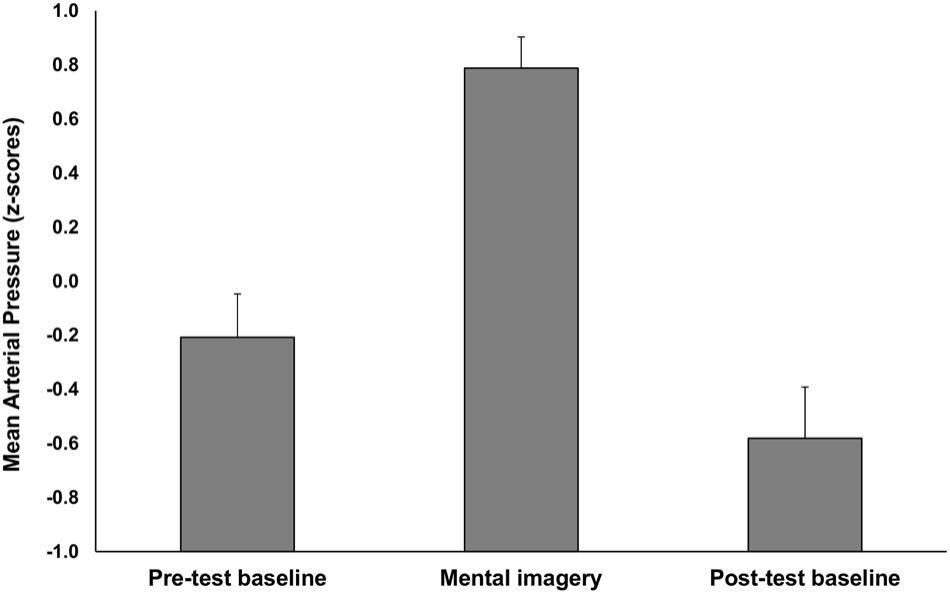
Changes in z-scored Mean Arterial Pressure after imagining negative risk consequences. Changes in blood pressure in pre-test and post-test baseline condition as compared to the mental imagery condition, *F*(2,42) = 58.687, *p* <. 001, η^2^ = .736. Error bars represent 95% confidence intervals.

Imagining the negative consequences of risk decreased positive affect (Δ*M* = -2.50, 95% CI [0.16, 4.86]) and increased negative affect (Δ*M* = 2.67, 95% CI [-0.17, 5.53]) as measured with the two PANAS scales, *F*(1,21) = 8.230, *p* = .009, η^2^ = .28 (see Fig. 3). The highest blood pressure was observed in the visualization phase (*M* = 0.79, 95% CI [0.67, 0.90]), *F*(2,42) = 58.687, *p* <. 001, η^2^ = .736 (see Fig. 4), in comparison to both baselines (the difference in mean stress level between baselines was not significant, *p* = .083). Moreover, we found a significant negative correlation between changes in positive affect and blood pressure, *r*(20) = -.37, *p* = .05. These results lead us to conclude that imagining the negative consequences of risk influences both psychological and psychophysiological measures of stress.

#### Multiple-step multiple mediator model of mental imagery, stress, risk perception and risk taking

**Fig 5:**
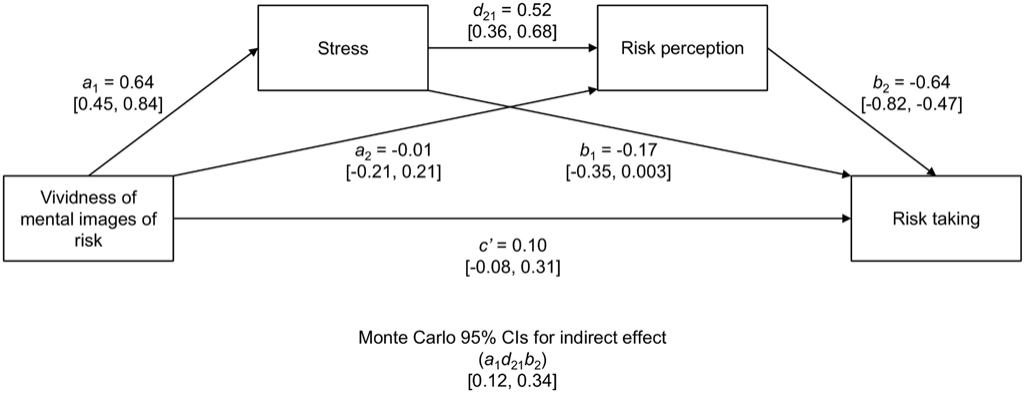
Multiple mediator model in Study 2. Unstandardized beta regression coefficients, corresponding to the serial multiple mediator model, linking vividness of mental images of risk (X) and risk taking (Y) via stress level (M_1_) and risk perception (M_2_). The model is controlled for individual differences in both stimulating and instrumental risk taking (SIRI). SIRI was negatively related only to risk perception, *r* = -.43, *p* = .048 (two-tailed). Lower and upper limit of 95% Monte Carlo confidence intervals are given in square brackets.

Similar to in Study 1, the serial multiple mediator model [21] with subjects and risk domains as crossed random-intercept effects was built to partition the total effect of intensity and vividness of mental images of risk consequences (X) on risk-taking propensity (Y) into direct and indirect (through mediating variables: M_1_—subjective experience of stress and M_2_—risk perception) components (see Fig. 5 for details including significant correlations between covariate variables and other measures). The model was controlled for individual differences in both stimulating and instrumental risk acceptance [20].

More vivid mental images of risk consequences led to higher stress (*a*_*1*_ = 0.64), which caused higher risk perception (*d*_21_ = 0.52). Similarly to Study 1, a higher perception of risk led to lower risk taking (*b*_2_ = -0.64), suggesting that serially related stress and risk perception measures mediated the relationship between mental images of risk and risk taking, 95% CI [0.12, 0.34]. In a mediation model such as this, the direct path between X and Y, while controlling for indirect effects, becomes insignificant (*c*’ = 0.10), which suggests the full mediation effect. That is, participants who imagined negative risk consequences more intensely and vividly were less likely to engage in risky behavior because their mental images of risk resulted in a stronger feeling of stress and led to assessing the risk as greater. Including mediator variables improved model fit, χ^2^(2) = 63.012, *p* <. 001, in comparison to the baseline model.

### Discussion

In Study 2, we demonstrated that imagining the negative consequences of risk enhances stress level and risk perception. Including these two measures as mediators improved overall fit of the model predicting the relationship between the mental representation of risk and risk taking. These results replicated findings obtained in the previous study. Moreover, we showed that the vividness and intensity of mental images or risk consequences were related to the subjective experience of stress: More vivid mental images of risk consequences led to more intense feelings of stress. Critically, an increase in the level of stress was observed not only in self-report measures but also in blood pressure changes. Such an effect appeared only in the imagery phase, during which participants were explicitly instructed to imagine risk consequences. This finding provides support for the causal relationship between the vividness of mental images of risk and the experience of negative emotions associated with stress. It may suggest that affect-laden imagery plays a substantial role in the process of risk perception/taking and should be considered as a crucial mediating psychological variable in more naturalistic risk scenarios where probabilities of the occurring events are not explicitly given [16].

In the previous section, we showed that negative emotional reactions such as stress play an important role as a mediator between affect-laden imagery, risk perception and risk taking. The vividness and emotional valence of mental images of risk evoke subjective feelings of stress that lead to higher risk perception and have an effect on risk taking. However, the strength of the identified relationships may depend on individual abilities to create vivid mental images. We addressed these issues in the next study. In Study 3 we investigated how individual differences in mental imagery moderate the relationship between risk perception and risk acceptance. Additionally, we introduced a within-subjects control condition to show the causal effect of imagining risk consequences on risky behavior.

## Study 3

People differ in the ease with which they create vivid mental images of various objects and situations [31,32]. Consequently, affect-laden mental images should evoke emotions that differ in intensity in people who vary in mental imagery ability. For instance, Holmes and Mathews [13] demonstrated that participants who were instructed to imagine unpleasant events instead of only listening to their description reported more anxiety, whereas Amit and Greene [33] found that individuals with a relatively high visual cognitive style made more affect-driven deontological judgments in comparison to individuals with a low visual cognitive style. Authors claim that measuring individual differences of affect-laden imagery could help better understand and interpret relationships between affective and cognitive psychological mechanisms. For example, Rottenstreich and Hsee [6] argue that vivid outcomes are more emotionally laden. This decreases sensitivity to changes in probabilities. We suggest that this process could be moderated by differences in affect-laden imagery abilities associated with visualizing risk consequences. Specifically, people who can create vivid mental images of risky behavior would be more prone to emotional influences and, hence, would be more likely to ignore factors like probabilities or risk assessments. On the other hand, people with a low mental imagery ability would use more cognitive information (e.g., risk assessment) to make their decisions.

In Study 3 we measured the objective intensity of mental imagery by means of registering brain activity to investigate whether it influences the strength of the relationship between risk perception and risk taking. Previous studies have established that there is an empirical association between the EEG activity and mental imagery [34,35,36,37,38]. We hypothesized that, for vivid imagers, the relationship between risk perception and risk taking would be weaker (risk perception would not be precisely translated into risk taking) than for non-vivid imagers who would more frequently and precisely use their risk perception as a cue to report their willingness to take risk. Furthermore, we aimed to replicate our previous findings and show that the intensity of mental images of risk consequences (here operationalized by the increase in brain activity) would contribute to a lower likelihood to take risk.

### Method

#### Participants

Seventeen undergraduate students (8 females) aged 23.6 on the average (*SD* = 3.2) participated in this study for credit points. All of them were right-handed according to the Edinburgh Handedness Inventory [39] and gave their informed consent to participate in the study. The participation in research was voluntary, anonymous and in agreement with the guidelines of the Ethical Committee.

#### Materials and apparatus

Participants were asked to listen to ten 30-second scenarios containing a short description of risky situations covering five risk domains (the same as in Study 1 and 2). Two independent scenarios were prepared for every risk domain. An additional risky scenario was used as training material to familiarize participants with the procedure (this scenario was not analyzed afterwards). One control scenario (a weather forecast), unrelated to risk, was used to register the EEG baseline data during imagining a neutral scene. This within-subjects control condition was presented twice and used to calculate a relative index of brain activity. Similarly to Study 2, each scenario was recorded with text-to-speech software to reduce the emotional effects of natural speech.

EEG data were recorded on the Biopac MP150 Data Acquisition System with Electrocap, keeping the International 10–20 System of electrode placement. Previous studies demonstrated that alpha activity in parieto-occipital areas is related to a state of relaxation, whereas the attenuation of alpha activity in these brain areas is generally associated with mental activity such as processing of sensory information or imagery [34,40]. For example, Davidson and Schwartz [35] showed that imagining flashing light led to an attenuated alpha rhythm in occipital lobes. The EEG study by Marks and Isaac [36] revealed attenuated alpha power during imagery in vivid-imagers in the left posterior quadrant of the cortex. According to the theoretical assumptions and empirical results, we chose six electrode sites covering the occipital (O1, O2) and parietal cortex (P3, P4) in both hemispheres. Prefrontal (Fp1, Fp2) electrodes served as control sites, areas in which we did not expect to observe activity related to mental imagery. A signal was sampled at 200 Hz with a 20,000 system amplification and an on-line high-pass filter (0.1 Hz). All electrode impedances were below the level of 5 kΩ. Both mastoids were used as on-line references.

#### Procedure

In order to measure individual differences in mental imagery, we adapted experimental protocols used in a previous EEG study, which aimed at showing the relationship between attenuated alpha activity in the parieto-occipital brain areas and imagery intensity [36,37,38]. The participants were seated in a dim laboratory room and were asked to relax for two minutes with their eyes closed. Next, one practice risk scenario was run (this scenario served as a training scenario to familiarize participants with the task and was not analyzed afterwards). The exact experimental procedure started with the baseline scenario (weather forecast), followed by ten risk scenarios presented in a pseudorandom order. At the end, baseline scenario (weather forecast) was presented once again. The beginning of every scenario was marked by a “beep” sound and ended with a phrase reminding the participants to imagine the negative consequences of the situation (“After hearing the signal, imagine all the negative consequences that might result from this situation”). Another “beep” sound marked the beginning of the 30-second visualization period. The next scenario started automatically after the 10-second inter-trial interval. Similarly to Study 2, participants did not verbally report their risk-related thoughts.

Declarative measures to diagnose risk perception and risk propensity were collected using a self-report questionnaire, which contained descriptions of all the scenarios presented in the EEG measurement stage. Participants were asked to rate: (1) the extent to which the situation presented was risky (i.e., risk perception, from 1 –“not risky at all” to 10 –“extremely risky”) and (2) their willingness to behave in the same way as the person described in the scenario (i.e., risk taking, from 1 –“definitely not” to 5 –“definitely yes”).

### Results

#### Overview of the results

The analysis of data collected in Study 3 was performed in two steps. First, EEG data were preprocessed to compute an indicator of the intensity of the mental imagery. Second, a moderation analysis was performed to show how the intensity of imagining the negative consequences of risk influenced risk-taking propensity as well as the strength of the relationship between risk perception and risk taking.

#### Data preprocessing

An EEG signal recorded from six electrodes (Fp1, Fp2, O1, O2, P3, P4) was filtered with EEGLab v6.03b software [41] at 35 Hz (low pass) and 0.1 Hz (high pass) cutoff frequencies. Afterwards, the EEG signal was divided into 2-sec epochs for each electrode site (Fp1, Fp2, O1, O2, P3, P4), scenario (2 baselines and 10 risky ones) and phase (listening or visualizing) separately. Epochs exceeding 70 μV were treated as artifacts and were subtracted from the data. This resulted in a rejection of 5% of the epochs. A Fast Fourier Transform was applied to each epoch in order to calculate an alpha band (8–13 Hz) power density (μV^2^/Hz). It has been suggested that alpha power is inversely related to brain activation [42]—the lower the alpha band power, the higher the activation in the specified region. It is also important to note that resting alpha activity shows large individual differences [43], which have to be accounted for to make inferences about task-specific EEG activity. In the present study, we were interested in showing how variation in EEG activity, which is related specifically to imagining risk consequences, would contribute to risk taking. Therefore, in order to adjust the absolute brain activity during mental imagery for individual differences in resting alpha band power, we used an index of relative alpha power—the Imagery Alpha Index (IAI). The IAI was calculated by dividing the averaged alpha power from both baselines by the averaged alpha power from the epoched risky scenarios for each risk domain (financial, health/safety, recreational, ethical, and social risk domain identified by Weber et al. [18]) and for each participant separately. That is, the averaged alpha band power from two baseline scenarios (weather forecast scenarios at the beginning and at the end of the study) was divided by alpha band power averaged over every two scenarios from each of the five risk domains. Similar relative indices have been previously used in EEG studies of hemispheric asymmetry [44,45,46]. The higher the IAI value, the higher the relative brain activity, suggesting more intense visualization processes. Data averaged this way were sent to a 2 (Hemisphere: left, right) x 3 (Electrode: Fp, O, P) x 5 (Scenario: financial, health/safety, recreational, ethical, and social) x 2 (Phase: imagining, listening) repeated measures analysis of variance.

#### EEG and behavioral data analysis

The statistical characteristics of the Imagery Alpha Index. All analyses were performed with the Greenhouse-Geisser sphericity correction. Data from one female participant were rejected due to excessive EEG signal distortions in the frontal electrodes. The significant main effect of Phase showed differences in the IAI between listening and visualizing stages, *F*(1, 15) = 45.16, *p* <. 001, η^*2*^ = .75. Neural activity in the mental imagery stage was higher (*M* = 1.04, 95% CI [1.03, 1.05]) than neural activity in the listening stage (*M* = 1.00, 95% CI [0.99, 1.02]).

Assuming that data from the listening stage were uninformative for hypothesis testing, further analyses were conducted for the visualizing stage data exclusively. A repeated measures ANOVA with 2 (Hemisphere) x 3 (Electrode) x 5 (Scenario) was carried out. As expected, the main effect of Electrode showed differences between the occipital (*M* = 1.06, 95% CI [1.5, 1.07]) and both the parietal (*M* = 1.03, 95% CI [1.02, 1.04]) and frontal lobes (*M* = 1.04, 95% CI [1.02, 1.05]) with the highest IAI values in the occipital sites, *F*(2, 30) = 11.49, *p* <. 001, η^*2*^ = .44. The interaction between Hemisphere and Electrode revealed higher IAI values for the left hemisphere, but only for the occipital sites, *F*(2,30) = 17.37, *p* <. 001; η^2^ = .54. This had been predicted on the basis of the results found in earlier studies [47]. The IAI means with 95% CIs for electrodes Fp1, P3 and O1 for the left hemisphere are 1.03 [1.02, 1.05], 1.01 [1.03, 1.06] and 1.07 [1.05, 1.08]. The IAI means with 95% CIs for the electrodes Fp2, P4 and O2 for the right hemisphere are 1.04 [1.02, 1.05], 1.05 [1.03, 1.06] and 1.05 [1.04, 1.07], respectively. Neither the significant main effect of Scenario nor the interaction effects between Scenario and other factors were found.

Moderation analysis. Moderation analysis was performed to investigate whether the strength of the association between risk perception and risk-taking measures depends on the intensity level of the mental imagery. Preliminary analyses showed that IAIs from the parietal and occipital electrode sites are highly correlated (Pearson’s *r* from. 43 to. 85; all *p*s <. 001). Therefore, in the subsequent steps of the analysis, we used only the IAI averaged across the EEG signal recorded from all the electrodes associated with mental imagery (O1, O2, P3, P4).

We modeled risk-taking propensity by fitting two models including subjects and risk domains as random-intercept effects ([23], similarly as in previous studies). In the first model, risk perception and mental imagery (average IAI) indicators were entered as fixed-effect predictors of risk-taking propensity (Y). As we predicted, participants who assessed the riskiness of the presented scenario as higher were also less likely to take risky behavior, *b* = -3,78, 95% CI [-5.88, -1.69]. Furthermore, in line with our previous results, a higher intensity of mental imagery led to a lower willingness to take risk, *b* = -25.36, 95% CI [-41.62, -9.13]. In the second model, we included the interaction term of these two fixed effects. As the data in Table 1 show, overall model fit as measured by log-likelihood significantly increased by introducing the interaction term, χ^2^(1) = 9.975, *p* = .002. Hence, the intensity of mental imagery was a significant moderator of the relationship between risk perception and risk taking.

**Table 1:** Moderated regression model for the association between risk perception (X) and risk taking (Y) with mental imagery intensity as a moderator.

Step	Predictors	*b*	*t*	95% CIs		df	Log-Likelihood	χ^2^	*p*
				LL	UL				
1	Risk perception	-3.78	-3.54	-5.88	-1.69				
	Intensity of mental imagery (mean IAI of all electrodes)	-25.36	-3.06	-41.62	-9.13	6	-93.084		
2	Risk perception x Intensity of mental imagery	3.34	3.29	1.34	5.32	7	-88.096	9.975	.002

**Fig 6:**
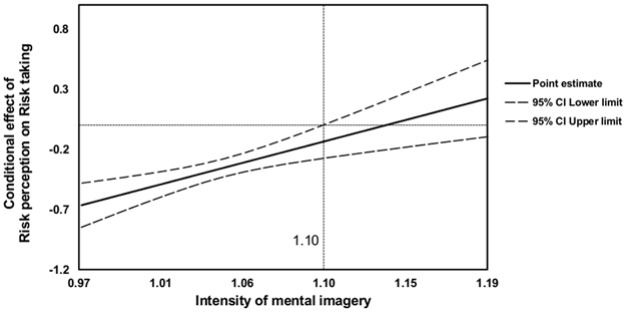
Johnson–Neyman regions of significance for the conditional effect of risk perception on risk-taking propensity in Study 3. The solid line plots the conditional effect estimates of risk perception on risk taking at values of the mental imagery intensity. The dashed lines represent 95% of the upper and lower bounds of confidence interval. For the point at which the mental imagery intensity is above 1.10, the relationship between risk perception and risk taking is not significant. Even though the lower bound of the confidence interval approaches zero, the effect does not become significant at the maximum value of the IAI (1.19) registered in this study.

To explore the character of the observed moderation effect, two types of conditional effect (simple slopes) analysis were performed. We investigated the relationship between risk perception and risk taking for three levels of moderator: mean and 1 SD above and 1 SD below mean. The strongest relationship was observed for the lowest IAI (*b* = -.52), a medium relationship was observed for the moderate level of the IAI (*b* = -.38) and the weakest relationship was observed (*b* = -.23) for high IAI participants (all *p*s <. 001). However, the pick-a-point approach presented above can be controversial due to the arbitrariness of choosing points (+/- 1SD) for a traditional simple slopes analysis. Thus, in the second conditional effect analysis, we used the Johnson–Neyman procedure for probing interaction [48]. In this technique, “regions of significance” are mathematically derived over the full spectrum of the moderator values for which the relationship between predictor and dependent variable is significant. If the confidence interval for the point estimate of the conditional effect does not contain a zero value, we can conclude that both predictor and dependent variable are significantly related to each other. As Fig. 6 shows, upper bound of 95% CI reaches zero at the moderator value of 1.10 (with the IAI ranging from 0.97 to 1.19). A result such as this suggests that, for high-imagery participants (with the IAI above the value of 1.10), the relationship between risk perception and risk taking becomes insignificant.

### Discussion

Different researchers have made an attempt to incorporate affect-laden imagery as an important factor in risk-taking behavior [10,49]. In Study 3, we once again provided evidence for the relationship between the intensity of mental images of risk and risk taking. Multiple regression analysis showed that participants’ willingness to take risk was negatively associated with increasing activity in brain regions associated with mental imagery. Importantly, introducing a control condition in which participants were instructed to visualize a scenario unrelated to risk supported our hypothesis that variations in the intensity and vividness of mental images of risk consequences influence willingness to engage in risky behavior.

What is more, we also showed that people’s ability to create vivid images of risk consequences (objectively measured by registering brain activity in the parieto-occipital regions) moderates the relationship between risk perception and risk taking. The more vivid and intense mental images of risk consequences were, the weaker and less precise was the relationship between risk perception and risk taking. To the best of our knowledge, there is little interest in considering this variable as a moderator of psychological processes related to risk perception.

Nevertheless, there is also a potential alternate explanation for the findings of this study. Specifically, the lack of additional measures of mental imagery has not allowed us to exclude the impact of other psychological processes, such as depth of processing, attention or task involvement, that might play an important role in risk perception and risk taking. For example, it could be not only mental imagery that is responsible for reduced willingness to take risk but also mental effort put into processing risky scenarios. That is, people can be less prone to take risk because of a general involvement in processing risk-related information (outcomes and probabilities) rather than because of intense mental images produced by a risky situation. To draw a clearer picture of this theoretical issue, it seems important to empirically examine whether these two processes (i.e., mental imagery and mental involvement) are related to one another. This methodological and conceptual issue should be addressed in future research. This would attempt to disentangle the effects of mental imagery on risk taking from other psychological factors.

One important methodological concern should be raised here. The design of our study included only one control scenario (i.e., a weather scenario) that was presented twice: at the beginning and at the end of the procedure. In this sense, control conditions were under-sampled in comparison to ten risky scenarios (however, it has to be emphasized that control scenarios were four times longer than each risky scenario resulting in a similar total time of visualizing phase). An alternative solution was to include ten control scenarios after each risky scenario. However, assuming that imagining risk consequences is related to stress reactions, EEG activity during built-in control conditions could have been distorted by these reactions (because of the post-stress recovery period [30]).

## General Discussion

### Overview of the results

The main goal of the present paper was to show that negative affect, driven by mental images associated with visualizing risk consequences, influences risk perception and subsequently impacts people’s willingness to engage in risky behavior. Another aim of this research project was to put forth a model that would best explain the propensity to engage in risk. Our results collected in the three studies suggest that risky behavior may be predicted most precisely when one takes into account both affective (subjective stress level) as well as more cognitive (risk perception) factors. This is consistent with the risk-as-feeling hypothesis [4].

Study 1 showed that the valence of risk-related thoughts evoked by imagining the consequences of risk may arouse subjective judgments of stress leading to enhanced risk perception and a lowered willingness to engage in risky behavior. We found that risk-related thoughts associated with imagining risk consequences are mostly negative. These negative images are related to higher risk ratings and stronger risk aversion. Study 2 replicated the basic outcomes of Study 1 but also showed a causal relationship between the vividness of risk-related thoughts and self-reported stress that influences both risk perception and risk taking. In Study 2, we used not only declarative measures of stress level but also indirect, physiological methods (changes in blood pressure). The results of this study suggested that visualizing negative aspects of risky behavior enhances feelings of stress as measured by means of blood pressure variations. We also confirmed that more intense self-reported stress induced by mental images leads to higher risk ratings and lower risk acceptance. In Study 1 and Study 2, people were asked to declare a valence and the vividness of mental images of risk. In contrast, in Study 3, we incorporated the method of electroencephalography (EEG) that allows brain activity to be registered in order to measure the intensity of risk visualizations. Again, we replicated the results of the two previous studies: More intense risk images, when controlling for an increase in risk perception, led to lower risk taking. In this study, we also demonstrated that individual differences in the ability to create vivid images of risk consequences moderate the causal relationship between estimating risk and being willing to engage in risky behavior. This result suggests that, when people experience strong affect, driven by visual images of risk, they do not tend to base their risky choices on risk estimations. This seems to be consistent with findings showing that emotions make people less sensitive to objective variations in probability [6]. For instance, recent studies that used a neuroimaging method have found hyperactivation of the amygdala-based emotional system and hypoactivation of the dorsal anterior cingulate cortex (dACC)-based analytical system for highly anxious people who made decisions under risk [50]. In this sense, it seems interesting to replicate our findings with the use of neuroimaging methods to provide further tests for the interaction of emotional and cognitive mechanisms in risk-taking behavior.

### Theoretical implications

The results of the research project presented in this paper seem to enrich our knowledge of the process of risky decision making in several ways. First, they suggest that, in natural situations associated with uncertainty, people might spontaneously imagine consequences of risky behavior which, in line with our results, evokes affective reactions. In accord with the priority heuristic [51], while making choices under risk, people concentrate first on negative information (possible losses) and only subsequently take into account positive outcomes (gains). Similarly, both behavioral studies [52] as well as neuroeconomic experiments [53] indicate that negative aspects of a decision problem weigh more than positive ones. Therefore, we assume in our model that in natural risky situations people imagine, first of all (or most vividly), the potential negative consequences of a particular risky behavior and that the strength of affect resulting from this visualization determines the subsequent relation between risk perception and risk taking. However, most risky situations elicit not only fear but also hope [54] and even if people focus on the negative aspects at first, they may also consider the positive outcomes of risk taking. In our subsequent studies, we plan to study not only the role of negative but also of positive images of risk in the process of risky decision making. Positive emotions should decrease risk assessments and increase people’s willingness to engage in risky behaviors.

Second, the series of studies that were presented in this paper suggest that affect plays a primary role when people consider their engagement in risky behavior. Moreover, the outcomes of Study 3 showed that people who create more vivid risk images (and probably are more prone to experience strong emotions when imagining these risk consequences) do not tend to base their decisions on cognitive risk estimations. On the other hand, under weaker negative affect, the association between risk perception and risk taking becomes stronger, i.e., the proneness to accept risk drops with higher risk ratings. These findings are consistent with theories showing the primacy of affect over cognition [55,56] but also with dual models of cognition that distinguish between emotional and analytical systems of mind [57,58]. In general, these models assume that affective System 1 is more automatic and faster than rational System 2 that is more effortful. If we apply this way of reasoning to the examination of decision making under risk, we may conclude that affect driven by imagining risk consequences precedes cognitive risk evaluation and, if strong enough, may lead to an immediate withdrawal from a risky situation.

Third, the results of our three studies confirmed that a decision to engage in risky behavior or not is causally determined by affective reactions to images of risk and subsequent risk perception. In particular, the model including both emotional and cognitive factors was better fitted to the data than other models not including these mediator variables. These two findings may be considered as a direct empirical test of the risk-as-feelings hypothesis [4] which assumes a core role for the interplay of both emotional and rational dimensions in the process of decision making under risk (see Fig. 1). Our research also confirmed the assumption made by the authors of the risk-as-feelings model that negative emotions experienced by decision makers in the face of risk depended greatly on mental imagery and particularly on the vividness of the mental images of the risk consequences.

### Limitations of the present studies and directions for future research

The results of our studies seem to shed a new light on the understanding of psychological processes that underlie risk-taking behavior. However, they also have limitations that we would like to raise.

In the present research we asked the participants to imagine negative consequences of different risk-related scenarios, but we have not directly controlled for the content of mental images they had produced (i.e., participants did not verbally report negative consequences they generated during the study). We designed our task so that it reflected naturalistic risk situations, in which people visualize decision consequences rather than verbalize them. It means, however, that we do not have evidence for what was the meaning of these images. Hence, a possible alternate explanation for results of Study 3 would be that participants differed not only in their ability to create vivid and intense mental images, but also in their overall involvement in the task. To overcome this limitation, future research ought to incorporate methods of measuring individual differences in mental imagery (e.g., Vividness of Visual Imagery Questionnaire [36]). This might help to disentangle the imagery effect from general involvement in task.

Another methodological problem arises from using single scales to evaluate stress, risk perception and risk taking measures. For example, a one-item scale that was used in all present studies could have increased random error and unsatisfactory reliability. However, one-item scales have widely been applied in research on risk perception [10,49]. It should be noted that all measures used in our study revealed satisfactory reliability (from. 68 to. 81) across risky scenarios and good validity demonstrated by significant correlations with the STAI and SIRI methods (see captions for Figs. 2 and 5).

One obvious limitation of studies reported in this paper is that we used self-report measures rather than measures of actual risk-taking behavior. As in other research on risk taking in natural environments, it is extremely difficult (or sometimes even unethical) to capture and control real instances of risky behavior (e.g., having unsafe sex with an unknown partner or driving after excessive alcohol consumption) as well as correlate them with psychophysiological measures (blood pressure and EEG). For these reasons, we attempted to study risk perception and risk-taking behavior using a questionnaire-based approach, which assumes that people have ability to introspect on their likely future behaviors and that their declarations are related to actual behavior. Nevertheless, self-report measures have been widely used in numerous studies on risk perception and risk taking (see [59] for a review) and significantly correlated with actual behavior [18,60,61]. It seems that both important and challenging would be to design future experiments verifying the role of affect in actual rather than declarative behaviors under risk.

### Conclusion and implications

Conclusions that may be drawn from our research seem to have not only theoretical but also practical implications. Many real-life decisions under uncertainty (how to invest money, whether to insure property, whether to have unsafe sex, whether to smoke, how fast to drive, etc.) may depend on the intensity and valence of images that result from thinking about risk. For example, based on our results, one could develop effective intervention programs that could reduce willingness to take risk in health domain, among young car drivers or adolescents taking drugs. Imagining vivid, personalized and negative consequences of risk could result in a higher perception of specific risks and, as a consequence, decreased risk taking.

The results of the present project suggest that under stress people do not tend to rely their choices on risk estimation but their actions are rather driven mostly by negative emotions. In consequence, they quickly withdraw from a risky situation and lose their chances. In practice, they may under diversify their financial portfolios (e.g., invest money in safe bonds exclusively) or overpay for insurance to avoid risk.

Summing up, our findings suggest that a better understanding of the risk-taking processes requires the examination of not only cognitive aspects of risk perception, but also affect-laden mental images that are elicited when people are faced with risk.

## Supporting Information

S1 Dataset Study 1 Dataset. (TXT)

S2 Dataset Study 2 Dataset. (TXT)

S3 Dataset Study 3 Dataset. (TXT)
